# The Suppression of Th1 Response by Inducing TGF-β1 From Regulatory T Cells in Bovine Mycoplasmosis

**DOI:** 10.3389/fvets.2020.609443

**Published:** 2020-12-02

**Authors:** Yamato Sajiki, Satoru Konnai, Shinya Goto, Tomohiro Okagawa, Kosuke Ohira, Honami Shimakura, Naoya Maekawa, Satoshi Gondaira, Hidetoshi Higuchi, Motoshi Tajima, Yuki Hirano, Junko Kohara, Shiro Murata, Kazuhiko Ohashi

**Affiliations:** ^1^Department of Disease Control, Faculty of Veterinary Medicine, Hokkaido University, Sapporo, Japan; ^2^Department of Advanced Pharmaceutics, Faculty of Veterinary Medicine, Hokkaido University, Sapporo, Japan; ^3^School of Veterinary Medicine, Rakuno Gakuen University, Ebetsu, Japan; ^4^Animal Research Center, Agriculture Research Department, Hokkaido Research Organization, Shintoku, Japan

**Keywords:** TGF-β1, *Mycoplasma bovis*, regulatory T cell, immunosuppression, cattle

## Abstract

Regulatory T cells (Tregs) regulate immune responses and maintain host immune homeostasis. Tregs contribute to the disease progression of several chronic infections by oversuppressing immune responses via the secretion of immunosuppressive cytokines, such as transforming growth factor (TGF)-β and interleukin-10. In the present study, we examined the association of Tregs with *Mycoplasma bovis* infection, in which immunosuppression is frequently observed. Compared with uninfected cattle, the percentage of Tregs, CD4^+^CD25^high^Foxp3^+^ T cells, was increased in *M*. *bovis*-infected cattle. Additionally, the plasma of *M*. *bovis*-infected cattle contained the high concentrations of TGF-β1, and *M*. *bovis* infection induced TGF-β1 production from bovine immune cells in *in vitro* cultures. Finally, we analyzed the immunosuppressive effects of TGF-β1 on bovine immune cells. Treatment with TGF-β1 significantly decreased the expression of CD69, an activation marker, in T cells, and Th1 cytokine production *in vitro*. These results suggest that the increase in Tregs and TGF-β1 secretion could be one of the immunosuppressive mechanisms and that lead to increased susceptibility to other infections in terms of exacerbation of disease during *M*. *bovis* infection.

## Introduction

Bovine mycoplasmosis caused by *Mycoplasma bovis* is prevalent in many countries, including Japan (1–4), and is characterized by chronic pneumonia, otitis, arthritis, and therapy-resistant mastitis (5–8). *M*. *bovis* has been well-documented as a causative agent of chronic pneumonia, and the exacerbation of disease is caused by co-infections with other agents (6, 7). However, the detailed mechanisms underlying the exacerbation of disease by co-infections during bovine mycoplasmosis have not been fully elucidated. The suppression of the immune response is frequently observed during *M. bovis* infection, leading to chronic progression. Several studies have demonstrated that *M. bovis* suppresses lymphocyte activities such as Th1 cytokine production and induces lymphocyte apoptosis *in vitro* (9, 10). In addition, our previous studies showed the association of immunosuppression by *M. bovis* with immunoinhibitory molecules, programmed death (PD)-1, PD-ligand 1 (PD-L1), and prostaglandin (PG) E_2_ (11, 12). PD-1/PD-L1 expression and PGE_2_ concentrations are increased in immune cells and the plasma of *M. bovis*-infected cattle, respectively (11, 12). The PD-1/PD-L1 pathway and PGE_2_ exert suppressive effects on Th1 cytokine production, such as interferon (IFN)-γ and tumor necrosis factor (TNF)-α, from bovine immune cells (13, 14). Therefore, the inhibition of the PD-1/PD-L1 pathway and PGE_2_ production *in vitro* activates *M. bovis*-specific Th1 responses (11, 12), which suggests that these inhibitory molecules might be involved in the immunosuppression during bovine mycoplasmosis. However, the detailed mechanisms of the immune suppression in this infection have not been fully elucidated.

Regulatory T cells (Tregs) constitute a subset of CD4^+^ T cells and are characterized by the expression of CD25, which is an interleukin (IL)-2 receptor α-chain, and forkhead box P3 (Foxp3), which is a transcription factor that is required for the development of Tregs (15). Tregs regulate the immune response by producing inhibitory cytokines, such as transforming growth factor (TGF)-β and IL-10, and maintain host immune homeostasis (16). Although Tregs are essential for host immune homeostasis, previous studies have reported the association of Tregs with the progression of chronic diseases by suppressing the immune response (17, 18). Tregs play an immunomodulatory role in several chronic infections, such as human immunodeficiency virus infection, hepatitis B virus infection, and hepatitis C virus infection (19–21). Several studies in the field of veterinary medicine have demonstrated that the immunomodulatory effects by Tregs are involved in the progression of chronic infections in cattle, such as Johne's disease and bovine leukemia virus infection (22–24). However, there are no studies demonstrating the association of Tregs with bovine mycoplasmosis.

The cytokine TGF-β exists in five isoforms, three of which (TGF-β1, TGF-β2, and TGF-β3) are expressed in mammals (25). TGF-β is a pleiotropic cytokine that is involved in both suppressive and inflammatory immune responses (26). Previous reports have shown that TGF-β–especially TGF-β1—plays an important role in immune modulation by regulating the activities of immune cells, including natural killer (NK) cells and T cells (27, 28). TGF-β controls innate immune responses such as NK cell cytotoxicity (29, 30). TGF-β also controls adaptive immunity by directly promoting the expansion of Treg cells and by inhibiting the generation and function of effector T cells and antigen presenting cells (31). In our previous study in cattle, we revealed that treatment with TGF-β reduces the expression of Th1 cytokines in T cells *in vitro* (23). However, the detailed immunosuppressive effects of TGF-β on bovine immune cells remain unclear.

In the present study, we examined the proportion of CD4^+^CD25^high^Foxp3^+^ cells and the concentration of plasma TGF-β1 in *M. bovis*-infected cattle by flow cytometry and enzyme-linked immunosorbent assay (ELISA), respectively. In addition, we analyzed the immunosuppressive effects of TGF-β1 on bovine peripheral blood mononuclear cells (PBMCs) *in vitro*.

## Materials and Methods

### Bacterial Strain

*M. bovis* strain PG45 (ATCC25523) was cultured in NK broth (Miyarisan Pharmaceutical, Tokyo, Japan) at 37°C for 72 h, collected by centrifugation, and washed with phosphate-buffered saline (PBS). Colony-forming units were counted using an NK agar plate (Miyarisan Pharmaceutical), followed by the resuspension of the bacteria in RPMI 1640 medium (Sigma-Aldrich, St. Louis, MO, USA) containing 10% heat-inactivated fetal bovine serum (Thermo Fisher Scientific, Waltham, MA, USA), 100 U/mL penicillin (Thermo Fisher Scientific), 100 μg/mL streptomycin (Thermo Fisher Scientific), and 2 mM L-glutamine (Thermo Fisher Scientific), and stored at −80°C until use.

### Bovine Samples

Blood samples derived from Holstein cattle were collected at several farms in Hokkaido, Japan. *M. bovis* infection was diagnosed clinically and microbiologically at Rakuno Gakuen University, Ebetsu, Japan, and Hokkaido University, Sapporo, Japan, as described previously (12). The blood collections of uninfected cattle, which had no history of *M*. *bovis* infection, were conducted at a *M*. *bovis* free farm, the Field Science Center for Northern Biosphere, Hokkaido University. The number of lymphocytes in the peripheral blood of uninfected and *M*. *bovis*-infected cattle was counted using a Celltac α MEK-6450 automatic hematology analyzer (Nihon Kohden, Tokyo, Japan). All experimental procedures using bovine samples were conducted following approval from the local committee for animal studies at Hokkaido University (approval No. 17-0024). Informed consent was obtained from all owners of cattle sampled in the present study.

### PBMC Culture

Buffy coat fraction was collected from blood samples by centrifugation (1,700 × g, 15 min, 25°C, without break). PBMCs were purified from collected buffy coat fraction by density gradient centrifugation (1,200 × g, 20 min, 25°C, without break) on 60% Percoll (GE Healthcare, Little Chalfont, UK). Then, collected PBMCs were washed 3 times with PBS by centrifugation (770 × g, 10 min, 25°C) and filtered through a 40-μm cell strainer (BD Biosciences, San Jose, CA, USA). PBMCs were stained with 0.4% Trypan Blue Stain (Thermo Fisher Scientific) and the number of the viable cells was counted using Countess II FL Automated Cell Counter (Thermo Fisher Scientific). In the PBMC cultures, PBMCs were cultured in RPMI 1640 medium as described above using 96-well plates (Corning, Corning, NY, USA) at 37°C under 5% CO_2_ atmosphere. PBMCs were incubated with live *M. bovis* at a multiplicity of infection (MOI) of 10:1. Culture supernatants were collected after 24 h, and TGF-β1 concentrations were measured by ELISA. To examine whether TGF-β1 suppresses Th1 responses in cattle, PBMCs were cultured with 10 ng/mL of recombinant human TGF-β1 (R&D Systems, Minneapolis, MN, USA) in the presence of 1 μg/mL of concanavalin A (Con A, Sigma-Aldrich). In accordance with manufacturer's protocols, recombinant human TGF-β1 was reconstituted in sterile 4 mM HCl (Kanto Chemical, Tokyo, Japan), and 4 mM HCl was used as a vehicle control in the experiments. Cells were harvested after 5 h, and CD69 expression was measured by flow cytometry. After 24h, culture supernatants were collected and IL-10 concentrations were determined by ELISA. After 72 h, culture supernatants were collected and the concentrations of IFN-γ and TNF-α were determined by ELISA.

### ELISA

PGE_2_ concentrations in plasmas were measured by Prostaglandin E_2_ Express ELISA Kit (Cayman Chemical, Ann Arbor, MI, USA), following the manufacturer's instructions. IFN-γ, TNF-α, and TGF-β1 concentrations in culture supernatants were measured by Bovine IFN-γ ELISA Development Kit (Mabtech, Nacka Strand, Sweden), Bovine TNF alpha Do-It Yourself ELISA (Kingfisher Biotech, St. Paul, MN, USA), and Human TGF-β1 DuoSet ELISA (R&D Systems) respectively, according to the manufacturers' protocols. As described in a previous paper with slight modifications (13), sandwich ELISA of IL-10 was performed using two antibodies; anti-bovine IL-10 (CC318; Bio-Rad, Hercules, CA, USA) as a capture antibody and biotinylated anti-bovine IL-10 (CC320; Bio-Rad) as a detective antibody. Briefly, a 96-well Maxisorp Nunc-Immuno Plate (Thermo Fisher Scientific) was coated overnight with CC318 diluted with carbonate-bicarbonate buffer (Sigma-Aldrich). After washing with PBS, blocking was performed by PBS-T (PBS containing 0.05% Tween 20) containing 0.1% bovine serum albumin (Sigma-Aldrich) for 1 h. After washing with PBS-T, the samples were incubated in the wells for 2 h. Following washing with PBS-T, diluted detective antibodies (CC320) were added to the wells and incubated for 1 h. After further washing with PBS-T, Neutra-Avidin-HRP (Thermo Fisher Scientific) was added and incubated for 1 h. Finally, the plate was washed with PBS-T and incubated with TMB One Component Substrate (Bethyl Laboratories, Montgomery, TX, USA), and absorbance was measured using MTP-900 (Corona Electric, Ibaraki, Japan). The results were calculated based on a standard curve (from 78 to 5,000 pg/mL) constructed using recombinant bovine IL-10 (Kingfisher Biotech).

### Flow Cytometry

Blood samples were treated with ACK buffer containing 8.26 mg/mL of NH_4_Cl, 1.19 mg/mL of NaHCO_3_, and 37.8 mg/mL of 2Na-EDTA (pH 7.3), and then washed twice with PBS. The staining of Tregs was then performed as described previously (23). Briefly, the cells were stained using the following reagents: FITC-conjugated anti-bovine CD4 antibody (CC8; Bio-Rad), Alexa Fluor 647-labeled anti-bovine CD25 antibody (IL-A111; Bio-Rad), FOXP3 Fix/Perm Buffer (BioLegend, San Diego, CA, USA), and PerCP/Cy5.5-conjugated anti-bovine Foxp3 antibody (FJK-16s; eBioscience, San Diego, CA, USA). PerCP/Cy5.5-conjugated rat IgG2a isotype control (eBR2a; eBioscience) was used as a negative control. After staining, the cells were analyzed by FACS Verse (BD Biosciences).

CD69 staining was performed as described in a previous paper (32). Briefly, collected cells were stained using the following antibodies: PerCP/Cy5.5-conjugated anti-bovine CD3 antibody (MM1A; Washington State University Monoclonal Antibody Center, Pullman, WA, USA), FITC-conjugated anti-bovine CD4 antibody (CC8), PE-conjugated anti-bovine CD8 antibody (CC63; Bio-Rad), and Alexa Fluor 647-labeled anti-bovine CD69 antibody (KTSN7A; Kingfisher Biotech). After staining, the cells were analyzed by FACS Verse.

### Statistics

Differences were assessed using the Mann-Whitney *U* test and the Wilcoxon signed-rank test. Correlations were analyzed using the Spearman correlation. A *p* value of <0.05 was considered to indicate statistical significance.

## Results

### Increase in CD4^+^CD25^**high**^Foxp3^+^ T Cells in *M. bovis*-Infected Cattle

**Figure 1:**
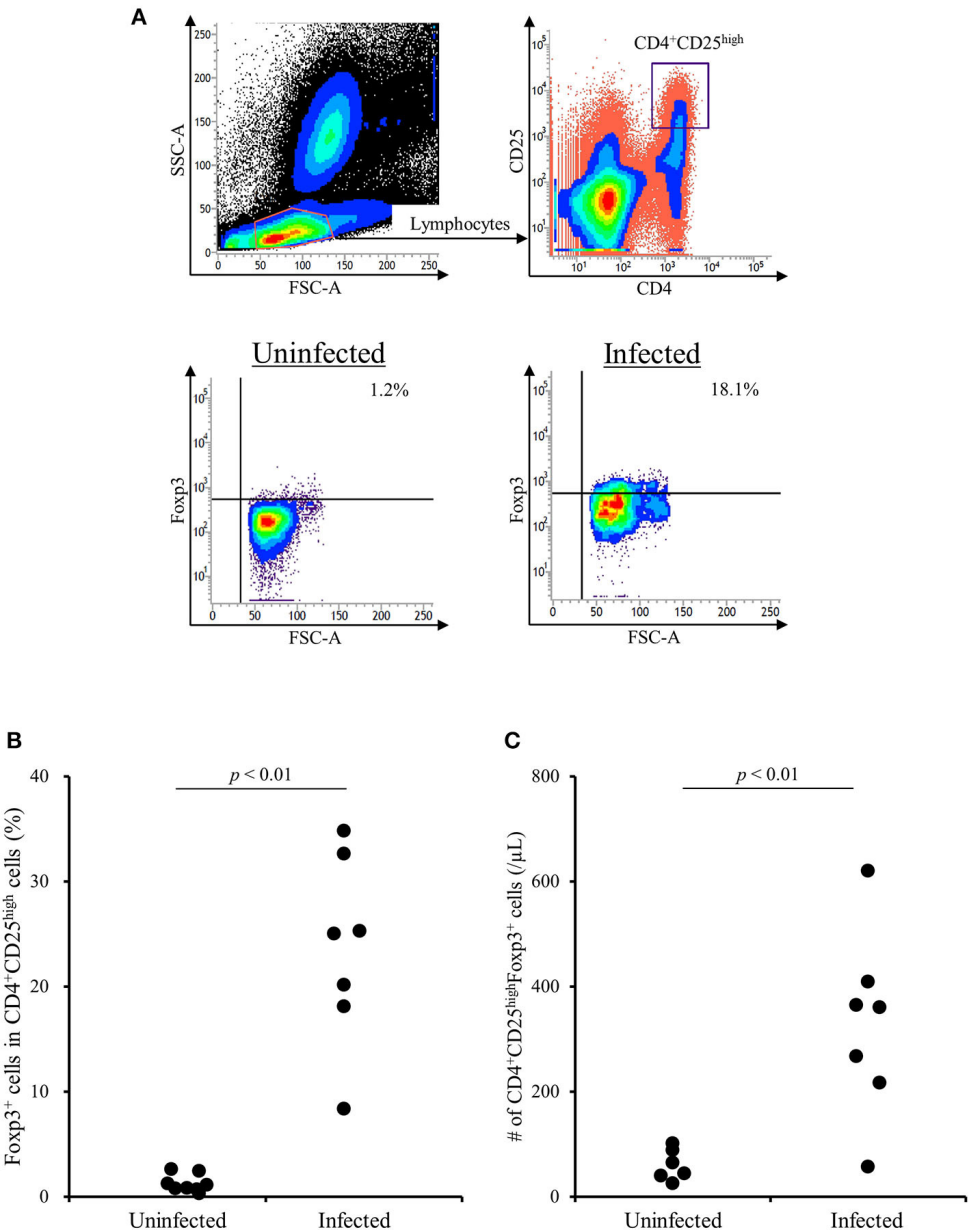
The increase of CD4^+^CD25^high^Foxp3^+^ T cells in *M. bovis*-infected cattle. **(A)** The gating strategy and representative plots for Treg staining. **(B)** The percentage of Foxp3^+^ cells in CD4^+^CD25^high^ T cells in *M. bovis*-infected (*n* = 7) and -uninfected (*n* = 8) cattle. **(C)** The number of CD4^+^CD25^high^Foxp3^+^ cells in *M. bovis*-infected (*n* = 7) and -uninfected (*n* = 6) cattle. **(B,C)** Statistical significance was determined by the Mann-Whitney *U* test. Uninfected, *M. bovis*-uninfected cattle; Infected, *M. bovis*-infected cattle.

**Figure 2:**
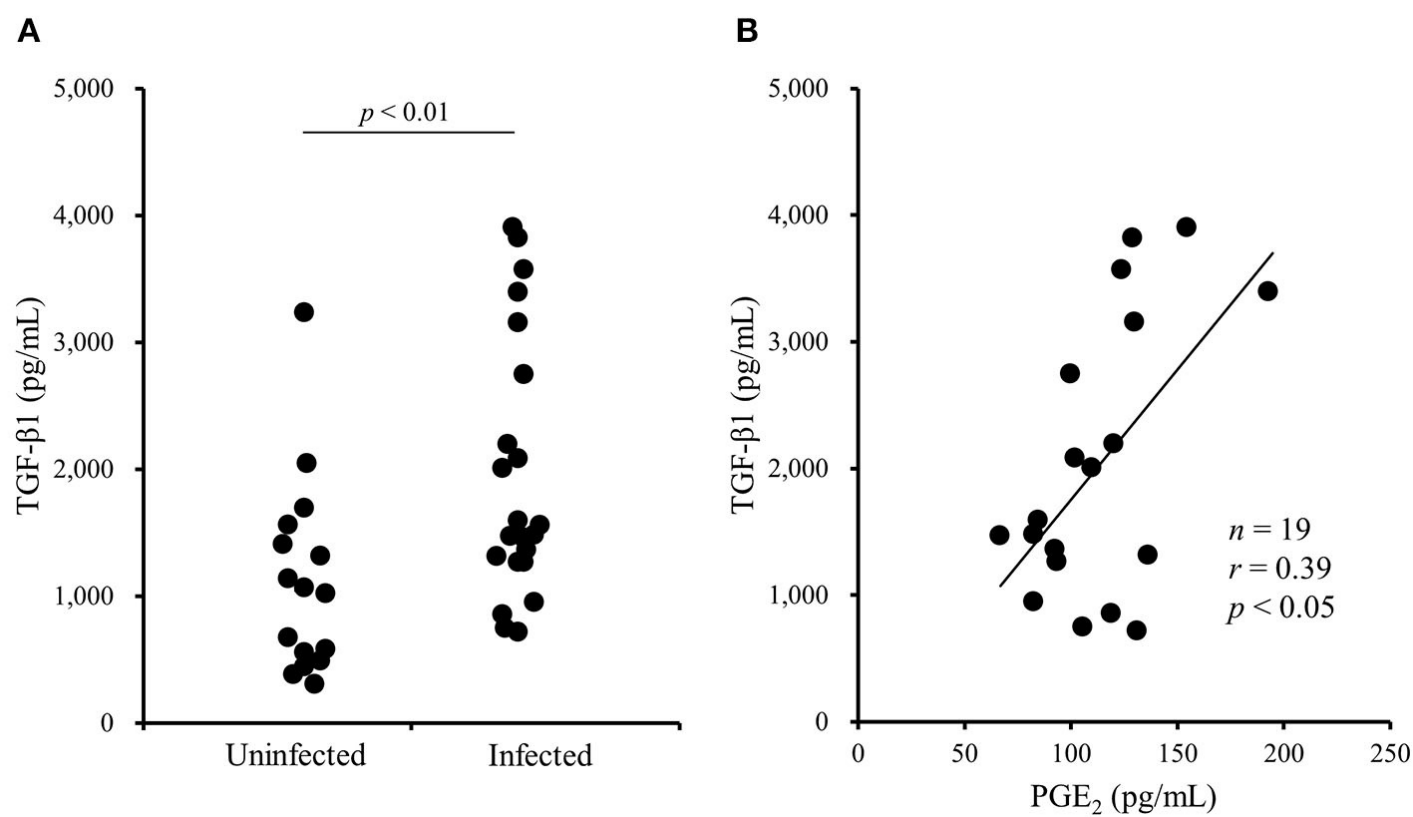
The increase of TGF-β1 concentrations in blood plasma of *M. bovis*-infected cattle. **(A)** TGF-β1 concentrations in the plasma from *M. bovis*-infected (*n* = 22) and -uninfected (*n* = 16) cattle were determined by ELISA. Statistical significance was determined by the Mann-Whitney *U* test. **(B)** The correlation between TGF-β1 and PGE_2_ concentrations in the plasma of *M. bovis*-infected cattle (*n* = 19). Correlation statistic was analyzed using the Spearman correlation.

A previous study has shown that the TGF-β1 secretion from Tregs reduces antiviral cytokine activities and the cytotoxicity of NK cells in cattle infected with bovine leukemia virus (23). However, the association of Tregs with other bovine chronic infections was still unclear. In the present study, we examined the percentage of Tregs in peripheral blood samples from cattle infected with *M. bovis*. Flow cytometric analysis revealed that the proportion of Foxp3^+^ cells in CD4^+^CD25^high^ cells was increased in *M. bovis*-infected cattle (Figures 1A,B, Table 1). The number of CD4^+^CD25^high^Foxp3^+^ cells in the peripheral blood was also increased in *M. bovis*-infected cattle (Figure 1C, Table 1). In addition, TGF-β1 concentrations in the plasma of *M. bovis*-infected cattle were significantly higher than those of cattle not infected with *M. bovis* (Figure 2A). Interestingly, TGF-β1 concentrations were positively correlated with PGE_2_ concentrations in the plasma of *M. bovis*-infected cattle (Figure 2B). Collectively, these results suggest the association of Tregs with *M. bovis*-infected cattle.

**Table 1:** The percentage and number of Tregs in *M*. *bovis*-infected and -uninfected cattle (raw data).

**Cattle**		**Foxp3^+^ /CD4^+^CD25^high^ (%)**	**CD4^+^CD25^high^Foxp3^+^/lymphocyte (%)**	**# of lymphocytes (/μL)**	**# of Tregs (/μL)**
Uninfected	U-1	1.23	0.0089	2,800	24.92
	U-2	0.84	0.0141	2,800	39.48
	U-3	0.78	0.0133	3,300	43.89
	U-4	2.61	0.0362	2,800	101.36
	U-5	0.71	0.0200	3,200	64.00
	U-6	2.46	0.0419	2,100	87.99
	U-7	0.32	0.0067	NA	NA
	U-8	1.14	0.0182	NA	NA
*M*. *bovis*- infected	M-1	34.79	0.0677	3,200	216.64
	M-2	24.99	0.1384	2,600	359.84
	M-3	25.27	0.0827	4,400	363.88
	M-4	8.33	0.0087	6,500	56.55
	M-5	32.61	0.0607	4,400	267.08
	M-6	18.09	0.1016	6,100	619.76
	M-7	20.13	0.1317	3,100	408.27

*NA, not available*.

### Induction of TGF- β1 Production by *M. bovis* Infection

**Figure 3:**
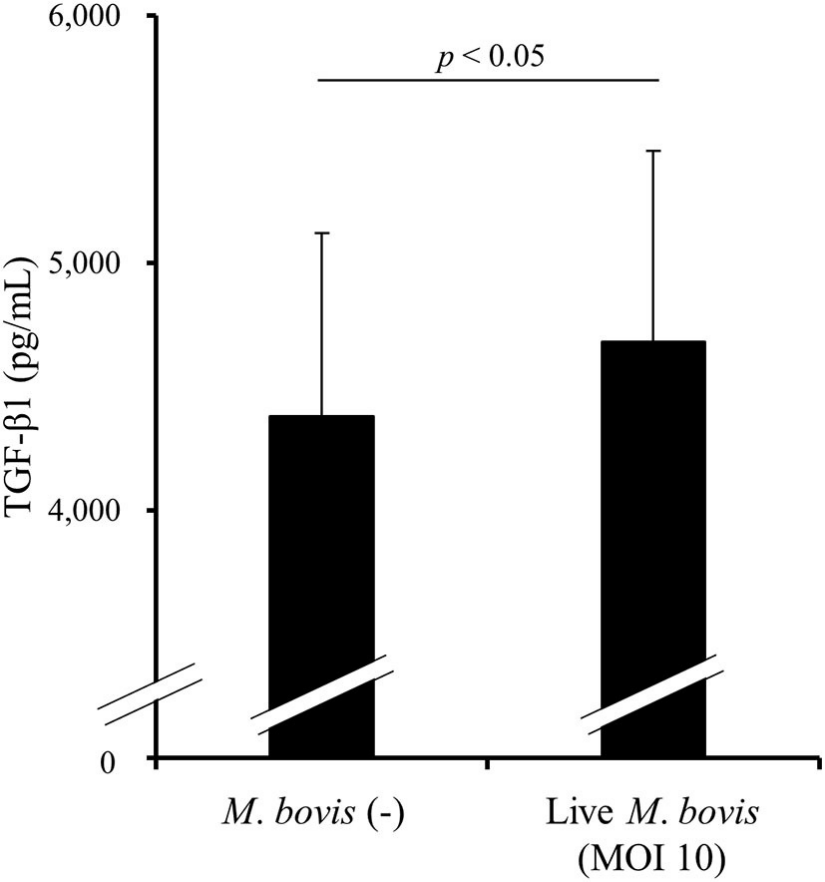
The induction of TGF-β1 production by *M. bovis*. PBMCs from uninfected cattle were cultured with live *M. bovis*, and TGF-β1 concentrations in culture supernatants were measured by ELISA (*n* = 8). Data are presented as means, and the error bars indicate standard errors. Statistical significance was determined by the Wilcoxon signed-rank test. MOI, multiplicity of infection.

To examine whether *M. bovis* directly induces TGF-β1 production during *in vitro* infection, PBMCs from uninfected cattle were cultivated with or without live *M. bovis* and TGF-β1 concentrations in culture supernatants were measured by ELISA. As shown in Figure 3, TGF-β1 concentrations were significantly increased in the cultures with live *M. bovis* when compared with that in the cultures without live *M. bovis* (Figure 3), suggesting that *M. bovis* can induce TGF-β1 production during its infection.

### Suppressive Effects of TGF- β1 on Th1 Responses Stimulated by Con A

**Figure 4:**
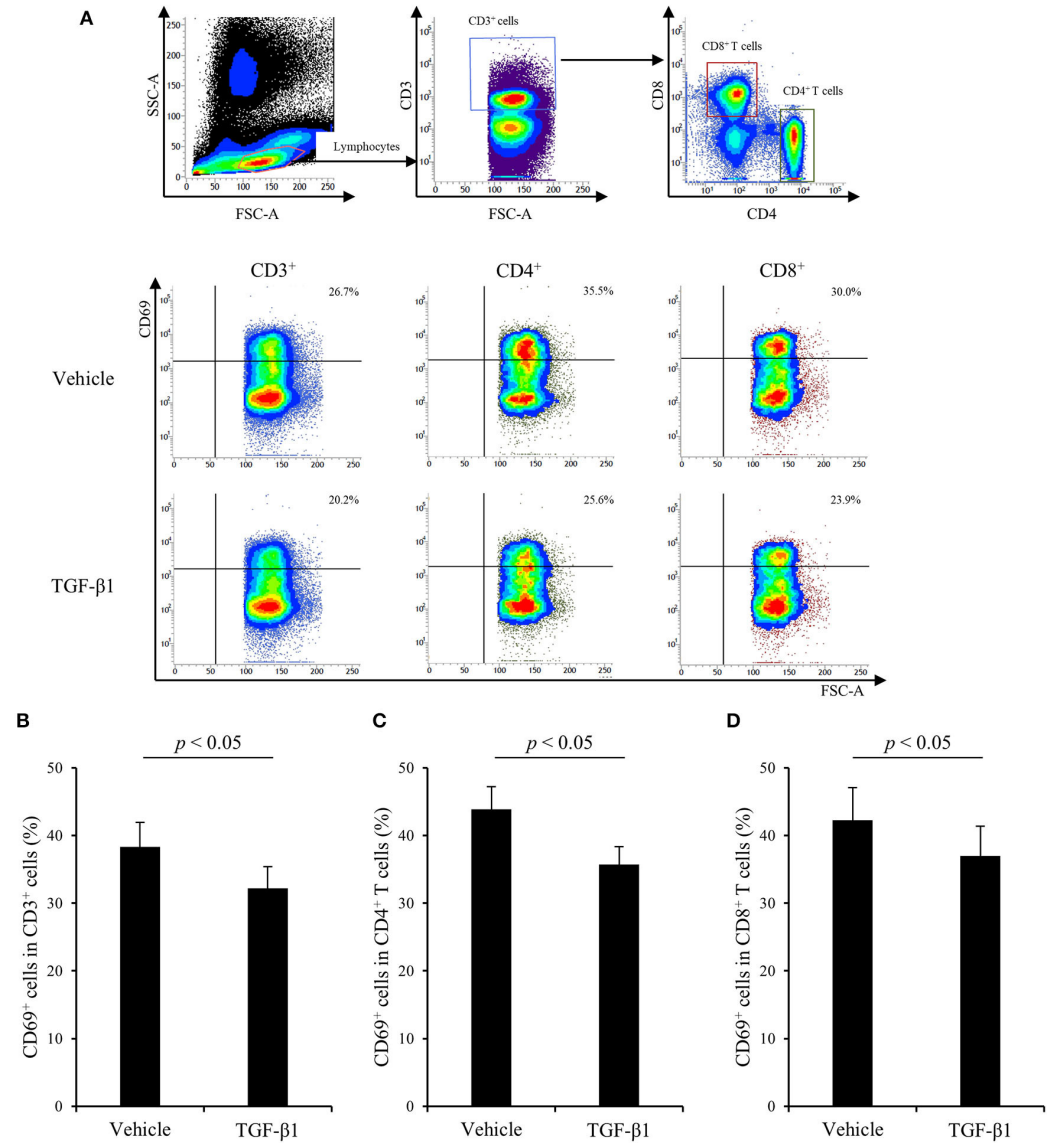
The effect of TGF-β1 on CD69 expression in cattle. **(A–D)** PBMCs from uninfected cattle (*n* = 7) were incubated with TGF-β1 in the presence of Con A. **(A)** The gating strategy and representative plots for CD69 expression. **(B–D)** CD69 expression in T cells **(B)**, CD4^+^ T cells **(C)**, and CD8^+^ T cells **(D)** were measured by flow cytometry. Data are presented as means, and the error bars indicate standard errors. Statistical significance was determined by the Wilcoxon signed-rank test.

**Figure 5:**
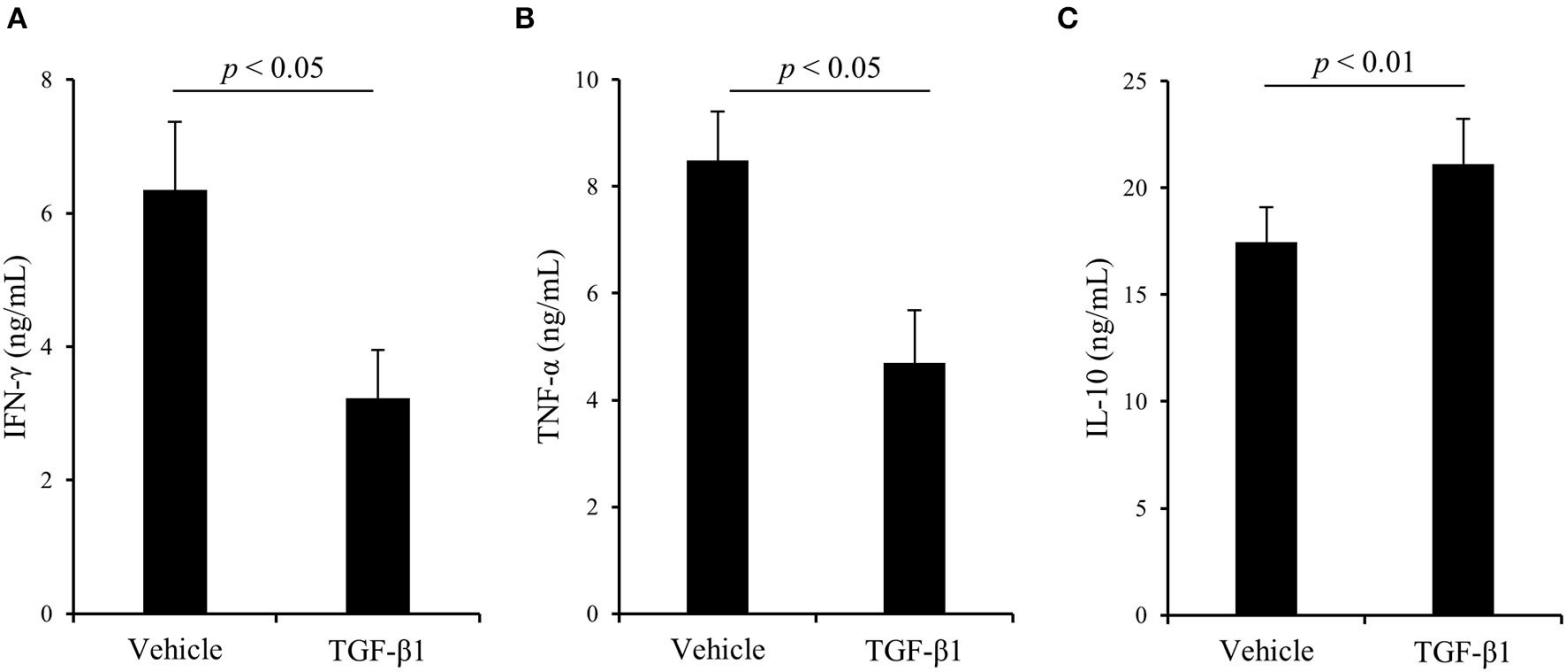
The effect of TGF-β1 on cytokine production in cattle. **(A–C)** PBMCs from uninfected cattle (*n* = 7) were incubated with TGF-β1 in the presence of Con A. After incubation, IFN-γ **(A)**, TNF-α **(B)**, and IL-10 **(C)** concentrations in culture supernatants were determined by ELISA. Data are presented as means, and the error bars indicate standard errors. Statistical significance was determined by the Wilcoxon signed-rank test.

Finally, to examine the effects of TGF-β1 on bovine Th1 responses in detail, PBMCs from uninfected cattle were cultured with TGF-β1 in the presence of Con A stimulation, and T-cell activation and cytokine production were measured by flow cytometry and ELISA, respectively. As shown in Figure 4, treatment with TGF-β1 reduced the expression of CD69, an activation marker of lymphocytes, in CD3^+^, CD4^+^, and CD8^+^ T cells *in vitro* (Figures 4A–D). Additionally, treatment with TGF-β1 suppressed IFN-γ and TNF-α production from bovine PBMCs *in vitro* (Figures 5A,B). In contrast, treatment with TGF-β1 induced the production of IL-10, an immunosuppressive cytokine, from bovine PBMCs *in vitro* (Figure 5C). Collectively, these results suggest that TGF-β1 has the suppressive effects on immune responses, especially the Th1 response, in cattle.

## Discussion

*M. bovis* has been shown to regulate bovine immune responses, including the induction of lymphocyte apoptosis and the suppression of Th1 cytokine production (9, 10). The effects of *M. bovis* on bovine immune response likely contribute to the chronic and nonresponsive progression of the disease. In the present study, we revealed the association of Tregs with *M. bovis* infection. We found that the percentage of CD4^+^CD25^high^Foxp3^+^ T cells was increased in cattle infected with *M. bovis*. Additionally, TGF-β1 concentrations in the plasma of *M. bovis*-infected animals were higher than in uninfected animals. Tregs are the suppressive subpopulation of CD4^+^ T cells and regulate immune responses by the secretion of inhibitory cytokines including TGF-β (16). Therefore, Tregs could be the source of TGF-β1 in the peripheral blood of *M. bovis*-infected cattle. These results suggest that the increase in Tregs could be one of the immunosuppressive mechanisms in *M. bovis*-infected cattle. Furthermore, the cultures using PBMCs of uninfected cattle revealed that treatment with TGF-β1 significantly downregulated Th1 responses, such as T-cell activation and Th1 cytokine production, *in vitro*. *In vitro* infection with *M. bovis* enhanced TGF-β1 production from bovine PBMCs. These data suggest that *M. bovis* promotes the secretion of TGF-β1 from host immune cells for its immune evasion, although the detailed mechanism remains unclear. Further experiments are required to elucidate the association of *M. bovis*-induced TGF-β1 with immunosuppression during *M. bovis* infection.

Recently, several studies have revealed the relationship between Tregs and other *Mycoplasma* infections in humans and mice (33, 34). Odeh and Simecka have demonstrated that CD4^+^CD25^+^ T-cell population is important to dampen inflammatory disease in *Mycoplasma pulmonis* infection of mice. However, the cell population does not contribute to persistence of infection (33). Guo and colleagues have shown that the association of an imbalance of circulating Tregs and Th17 cells with the deterioration of patients with *Mycoplasma pneumoniae* pneumonia. Although the Th17/Treg ratio is significantly higher in the patients with refractory *M*. *pneumoniae* pneumonia in comparison with healthy control, there is no significant difference with the frequencies of Tregs and the levels of TGF-β1 in the patients with refractory *M*. *pneumoniae* pneumonia (34). These studies suggest that Tregs might not be involved in the chronic progression of *Mycoplasma* infections. However, to the best of our knowledge, this is the first study to show the association of Tregs with cattle infected with *M*. *bovis*. Additionally, it is still unclear whether the increase in Tregs and Treg-derived cytokines truly leads to the progression of *M*. *bovis* infection. Therefore, future experiments are necessary to further elucidate roles of Tregs in the pathogenesis of *Mycoplasma* infections including *M*. *bovis* infection.

PGE_2_ is an inflammatory mediator derived from arachidonic acid by several enzymes, such as cyclooxygenase (COX)-1 and COX-2 (35). PGE_2_ inhibits the activity of immune cells, such as T cells, dendritic cells, and NK cells (36). Our previous studies have revealed that the immunosuppressive effects of PGE_2_ contribute to the disease progression of several chronic infections in cattle, including *M. bovis* infection (12, 14, 37). Interestingly, in this study, TGF-β1 concentrations were positively correlated with PGE_2_ concentrations in the plasma of *M. bovis*-infected cattle. Previous studies on human research have described that treatment with TGF-β1 *in vitro* induces PGE_2_ production from several cell types including CD4^+^ T cells (38, 39). Our previous and present studies have shown that *M. bovis* upregulates PGE_2_ and TGF-β1 production from bovine immune cells (12). Hence, PGE_2_ upregulation in *M. bovis*-infected cattle might be caused via TGF-β1 production by *M. bovis*. Conversely, previous studies have shown that PGE_2_ induces *Foxp3* gene expression and enhances the induction and differentiation of Foxp3^+^CD25^+^CD4^+^ Tregs (40–42). Our previous study has shown that treatment with PGE_2_ upregulates *TGF-*β*1* and *Foxp3* expression in bovine immune cells (37). Thus, the cross-interaction between *M. bovis*-induced TGF-β1 and PGE_2_ might have the potential for exacerbating immune suppression during *M. bovis* infection.

Here, we demonstrate the association of Tregs with *M. bovis*-infected cattle. Additionally, *M. bovis* promotes the secretion of TGF-β1, which has a suppressive effect on immune responses, especially Th1 immune responses, in cattle. These findings might contribute to the increase in susceptibility to other infections regarding the exacerbation of disease because co-infection with other bacteria and viruses has been frequently observed during *M. bovis* infection (6, 7). Further experiments are required to elucidate the influence of *M. bovis*-induced TGF-β1 in the exacerbation of disease by co-infections during the bovine mycoplasmosis. Presently, *M. bovis* infection is spreading globally (1–4), and there are no effective vaccines due to the immunosuppression caused by *M. bovis*. Therefore, a greater understanding of the immunosuppressive mechanism is necessary to develop a novel control strategy of bovine mycoplasmosis. Our results could contribute to the development of an effective control method against this infection.

## Data Availability Statement

The original contributions presented in the study are included in the article/supplementary material, further inquiries can be directed to the corresponding author/s.

## Ethics Statement

The animal study was reviewed and approved by Local committee for animal studies at Hokkaido University (Approval No. 17-0024). Informed consent was obtained from all owners of cattle sampled in the present study. Written informed consent was obtained from the owners for the participation of their animals in this study.

## Author Contributions

SK, SM, and KOha: designed the work. YS, SGot, TO, HS, KOhi, and NM: performed the experiments. YS, SK, SGot, TO, and KOhi: acquired, analyzed, and interpreted the data. SGon, HH, MT, YH, and JK: provided blood samples and laboratory reagents. YS and SK: wrote the manuscript. SK, TO, NM, SM, and KOha: revised the manuscript. All authors reviewed and approved the final manuscript.

## Conflict of Interest

The authors declare that the research was conducted in the absence of any commercial or financial relationships that could be construed as a potential conflict of interest.
